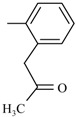# Multidirectional Efficacy of Biologically Active Nitro Compounds Included in Medicines

**DOI:** 10.3390/ph11020054

**Published:** 2018-05-29

**Authors:** Dorota Olender, Justyna Żwawiak, Lucjusz Zaprutko

**Affiliations:** Department of Organic Chemistry, Pharmaceutical Faculty, Poznan University of Medical Sciences, Grunwaldzka 6, 60-780 Poznan, Poland; dolender@ump.edu.pl (D.O.); zaprutko@ump.edu.pl (L.Z.)

**Keywords:** nitro group, nitro compounds, drugs with the nitro group, veterinary medicines with the nitro group

## Abstract

The current concept in searching for new bioactive products, including mainly original active substances with potential application in pharmacy and medicine, is based on compounds with a previously determined structure, well-known properties, and biological activity profile. Nowadays, many commonly used drugs originated from natural sources. Moreover, some natural materials have become the source of leading structures for processing further chemical modifications. Many organic compounds with great therapeutic significance have the nitro group in their structure. Very often, nitro compounds are active substances in many well-known preparations belonging to different groups of medicines that are classified according to their pharmacological potencies. Moreover, the nitro group is part of the chemical structure of veterinary drugs. In this review, we describe many bioactive substances with the nitro group, divided into ten categories, including substances with exciting activity and that are currently undergoing clinical trials.

## 1. Introduction

Drugs exert a local action, or, after being absorbed into the body, a general effect. The chemical structure of the active substance is fundamental because of its therapeutic effect on the body. As a result of its connection with specific structures (multiparticulates—receptor), various types of transformations occur, which consequently leads to particular pharmacological effects. Medicines containing the nitro group in their structure constitute a huge family, which is diverse in terms of pharmacology and chemical structure. The nitro group is a chemical functional group containing two oxygen atoms that are bound to a nitrogen atom, which connects the group to the rest of the molecule. With regard to the chemical structure, it should be noted that the nitrogen atom is characterized by a large deficit of negative charge. Therefore, on an aromatic ring, it has a strong electron withdrawing effect that deactivates the ring, because the resonance effect causes the “pull” of electrons from the cyclic aromatic structure. Sometimes the presence of the nitro group may be responsible for the toxicity of certain drugs.

In biological systems, the nitro group undergoes enzymatic reduction, which can take place by both a one- or two- electron mechanism. Sequential two-electron reduction of the NO_2_ group gives amines via nitroso and hydroxylamine intermediates. The nitroaromatics and amines remains unchanging, but sometimes the nitroso and hydroxylamine intermediates can react with biomolecules to produce compounds having undesired effects. A one-electron reduction of the nitro group produces a nitro radical anion, which is unstable. Under aerobic conditions, it is reoxidized back to the nitro group by molecular oxygen, which is in turn reduced to form a reactive superoxide anion. This process is named the “futile cycle.” In many cases, it is connected with the toxicity of compounds having NO_2_ group. Also, these reactive species are connected with the acting of nitroso compounds as a pro-drug. The wide range of applications suggests, therefore, that nitro drugs are an essential part of chemotherapy. 

## 2. Drugs Used in Cardiovascular Diseases

Nitro compounds are used in the treatment of many diseases, including hypertension, coronary artery disease, and heart failure, and in the prevention of stroke in atrial fibrillation and thromboembolism. Drugs that are used in cardiovascular diseases include organic nitrates that are characterized by C-ONO_2_, β-blockers, calcium channel blockers, anticoagulants, fibrinolytics, omega 3-fatty acids, and free radical scavengers [1,2]. 

### 2.1. Organic Nitrates

**Figure 1:**
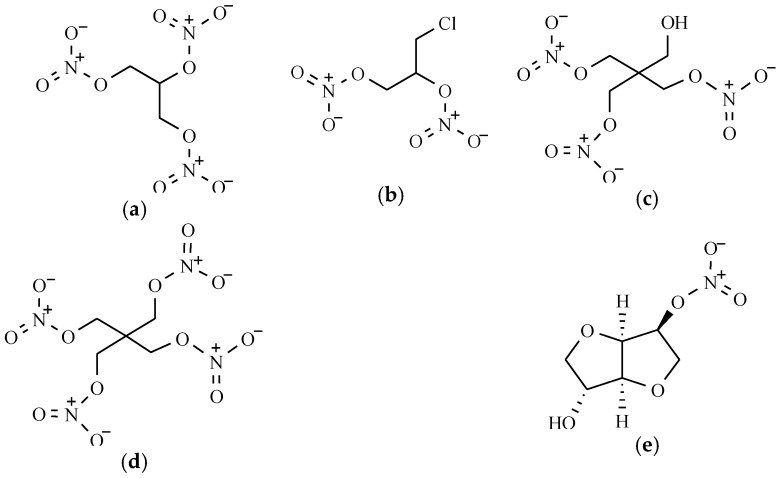
The chemical structure of some organic nitrates: (**a**) Nitroglicerinum; (**b**) Clonitrate; (**c**) Pentrinitrol; (**d**) Nitropentaerythritol; and (**e**) 5-Isosorbide mononitrate.

Preparations of nitroglycerin (trinitrate glycerol) from the group of organic nitrates are mainly used. These are characterized by very high membrane permeability and very low stability [3]. This group also includes such substances, as: clonitrate, trinitrotriethanolamine diphosphate, pentrinitrol (Petrin) and nitropentaerythritol. To avoid the accidental explosion, nitropentaerythritol is combined with lactose and d-glucitol derivatives. Isosorbide dinitrate is used in the treatment and the prophylaxis of angina pectoris and is partially metabolized to weaker but much longer acting 5-isosorbide mononitrate. 5-Isosorbide mononitrate is also known as a separate formulation [2,3] (Figure 1).

#### Organic Nitrates’ Mechanism of Action

Organic nitrates act as prodrugs for nitric oxide and are used to treat or prevent acute attacks of angina pectoris. These agents have been in full-scale use for many decades and have not been implicated in causing serum enzyme elevations or clinically apparent liver injury. Organic nitrates do not release NO in a simple way. Very often, nitrate groups react with enzymes and intracellular -SH groups cause a reduction in the nitrate groups to NO or to S-nitrosothiol, which then undergoes reduction producing NO. Nitric oxide activates smooth muscle soluble guanylyl cyclase (GC) to form cGMP. Increased intracellular cGMP inhibits calcium entry into the cell, thereby decreasing intracellular Ca concentrations and causing smooth muscle relaxation. NO also influences K^+^ channels, which leads to hyperpolarization and relaxation. Finally, when acting through cGMP, NO can stimulate a cGMP-dependent protein kinase that activates myosin light chain phosphatase, which is the enzyme that dephosphorylates myosin light chains, which leads to relaxation [4].

The pharmacological action of nitrate involves the vasodilation of venous and arterial vessels (including coronary arteries), thereby reducing pre- and afterload of the left ventricle, as well as improving coronary blood flow. Smooth muscle is probably due to the activation of guanylate cyclase and increasing cyclic GMP levels that are responsible for NO formed from an organic nitrate. For stable coronary heart disease, myocardial infarction acute heart failure or hypertensive crisis, preparations of nitroglycerin can be used. Nitrates are typically given sublingually and have a rapid onset of action and a somewhat short duration of action. Frequently appeared side effects of nitrates are a headache (caused by cerebral vasodilation) and cutaneous flushing. Other side effects include hypotension and reflex tachycardia. Excessive hypotension and tachycardia can worsen angina by increasing the oxygen demand. 

### 2.2. β-Blockers Others Cardiovascular Drugs

In the long-term treatment of angina, nicorandil, which is a derivative of nicotinic acid amide, and nipradilol is one of the β-blockers from the benzopyran group are also helpful [3].

### 2.3. Calcium Channel Blockers 

The nitro drugs class also includes nifedipine derivatives, which in chemical terms are derivatives of 1,4-dihydropyridine (Table 1). The presence of the nitro group in their structure sensitizes nifedipines to light [1]. It has been found that the best therapeutic properties belong to compounds containing a hydrogen atom on the nitrogen atom in the dihydropyridine ring and where methyl or cyano groups are present in positions 2 and 6. Moreover, the ester group should be attached to the C-3 and C-5 position, and an aromatic substituent should be on the C-4 atom of this ring. 

#### Nifedipine Derivatives’ Mechanism of Action

The pharmacological activity mechanism of nifedipine derivatives categorizes this class of compounds as selective calcium channel blockers which can affect different aspects of the body to serve specific purposes based on a person’s unique health conditions. Calcium has several effects on the body, one of which is triggering heart muscle contraction by blocking calcium channels in vascular smooth muscle, nifedipine prevents them from opening during stimulation [5]. Even in small doses, it inhibits the penetration of calcium ions into smooth muscle cells throughout the vascular system, including renal and cerebral vessels. This reduces the concentration of Ca^2+^ in the cytosol, thereby decreasing the strength of the muscle contraction [6]. Nifedipine inhibits voltage-dependent l-type calcium channels, which leads to vascular (and other) smooth muscle relaxation, and it has an anti-anginal and antihypertensive effect. 

Vasodilation, followed by a baroreceptor-mediated increase in sympathetic tone then results in reflex tachycardia [7].

### 2.4. Treatment of Thromboembolic Disease 

**Figure 2:**
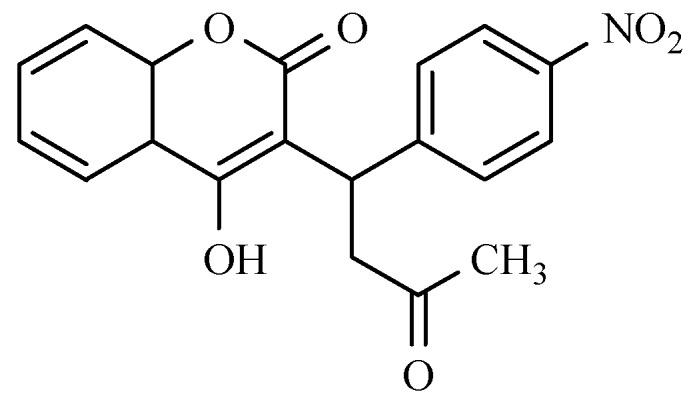
The chemical structure of acenocoumarol.

In the prevention and treatment of thromboembolic disease acenocoumarol, a 4-hydroxycoumarin derivative is applied (Figure 2) [8]. Acenocoumarol is a mono-coumarin derivative with a racemic mixture of R (+) and S (−) enantiomers. 

This drug is a vitamin K reductase antagonist. It inhibits the biosynthesis of clotting factors, including prothrombin. Acenocoumarol is one of the more frequently used oral anticoagulant therapies. Oral anticoagulant therapy is indicated for many diseases, including the prevention of stroke in atrial fibrillation, mechanical heart valve prosthesis and some valvular diseases, deep vein thrombosis, and pulmonary embolism. Acenocoumarol is effective and safe for all age groups [9].

## 3. Anxiolytics

Benzodiazepines are the most frequently represented pharmacologically active substances with anxiolytic activity with the nitro group. They are mostly a series of derivatives of 1,4-benzodiazepin-2-one. The presence of specific substituents is important for the scope of their activity. The nitro group or halogen (Cl, Br) at the 7-position, enhances the therapeutic action of the drug, and it is relatively strongly hypnotic. Similarly, the presence of the methyl group at the 1-position increases its activity, while an ethyl substituent reduces this effect. An increase in pharmacological effect also occurs in the event of hydroxyl moiety at the 3-position and the halogen atom in the ortho position of the phenyl substituent at C-5.

**Figure 3:**
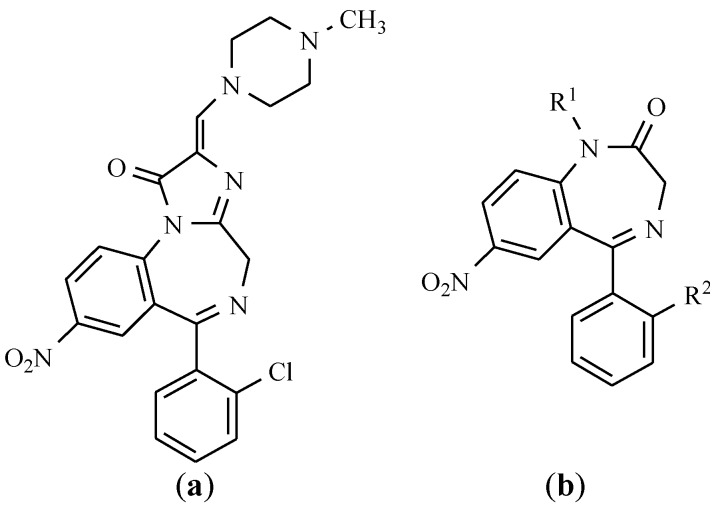
The chemical structure of benzodiazepine derivatives: (**a**) Loprazolam; (**b**) Nitrazepam: R^1^, R^2^ = H, Clonazepam: R^1^ = H, R^2^ = Cl, Flunitrazepam: R^1^ = CH_3_, R^2^ = F, Nimetazepam: R^1^ = CH_3_, R^2^ = H.

Benzodiazepines that are used in therapy include the following compounds with the nitro group (Figure 3):

- Nitrazepam is used in short-term insomnia, and as adjunctive therapy in the treatment of epilepsy and preparation for surgery (a day before surgery overnight) [10].
- Flunitrazepam is a fluorine-benzodiazepine derivative with a strong sedative and hypnotic activity being applied for the treatment of sleep disorders, including premedication as an agent in anesthesia and intensive care [11].
- Clonazepam is a chloro derivative of nitrazepam, which is characterized by anti-convulsant activity. Clonazepam, more than other benzodiazepines, is of benefit in the treatment of some types of myoclonus. Its mechanism of action is to facilitate GABAergic transmission in the brain directly on benzodiazepine receptors [12]. It is one of the most effective antiepileptic drugs.
- Nimetazepam is the N-methyl derivative of nitrazepam with a sedative and hypnotic effect. This drug is not registered in Poland.
- Loprazolam is a tricyclic derivative of imidazo-1,4-benzodiazepine, and it mainly exhibits hypnotics, anxiolytics, sedatives, anticonvulsants, and muscle relaxants effects [13].

In addition to anxiolytic activity, these compounds also have sedative, anticonvulsant, and muscle relaxant effects. They cause polysynaptic reflexes by inhibiting the spinal cord, but long-term use can lead to a state of drug dependency. All of these actions are the results of potentiating neuronal inhibition mediated by specific receptors. Benzodiazepine forms an integral part of the GABA_A_-ionophore (channel) chloride complex. 

## 4. Drugs Used in Parkinson’s Disease

**Figure 4:**
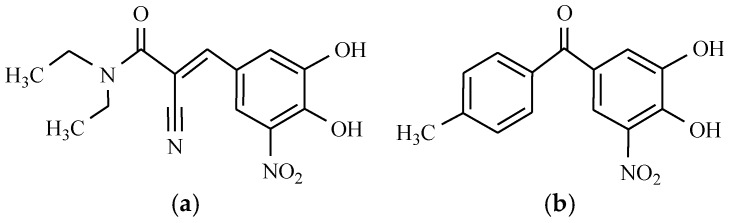
Parkinson’s disease drugs: (**a**) Entacapone; and (**b**) Tolcapone.

Parkinson’s disease treatment works to restore the neurohormonal balance in the extrapyramidal system, which can be achieved by increasing the dopamine concentration using levodopa (dopamine precursor) agents that inhibit the metabolism of dopamine or drugs, which increase the release of dopamine from the synaptic granules. Pharmaceuticals with the nitro group, which increase the concentration of dopamine, including entacapone and tolcapone [1] withdrawn from the treatment due to the large hepatotoxicity. In these compounds, nitro group is connected with the benzene ring (Figure 4).

Entacapone is structurally and pharmacologically similar to tolcapone, but unlike tolcapone, it is not associated with hepatotoxicity. Entacapone is used in the treatment of Parkinson’s disease as an adjunct to levodopa/carbidopa therapy [14]. These two drugs are selective and potent catechol-O-methyltransferase (COMT) inhibitors that slow down the metabolism of levodopa, thus prolonging its effects. Entacapone represents one of the cornerstones of therapy for Parkinsons’ disease, and which is particularly useful in motor fluctuations. The main side effects usually consist of dyskinesia and gastrointestinal symptoms, and although adverse cardiovascular effects have been identified, the drug has so far demonstrated an acceptable safety profile. 

However, adjunctive therapy with tolcapone can significantly reduce the dose of levodopa that is required for illness treatment. Moreover, the use of tolcapone significantly reduces wearing off and on-off periods in fluctuating patients and improves “on” time in patients with stable disease. Tolcapone produces the expected dopaminergic side effects, such as a headache, nausea, insomnia, and diarrhea. Fortunately, these undesirable effects are mild and as a rule do not result in the discontinuation of therapy [15,16].

## 5. Drugs Used in Peptic Ulcer

**Figure 5:**
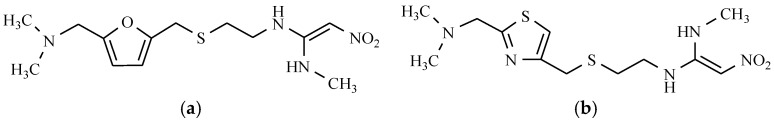
Antiulcerative drugs: (**a**) Ranitidine; and (**b**) Nizatidine.

Antiulcer drugs containing the nitro group in their structure, in terms of the mechanism through which they act in the organism, are H_2_ receptor antagonists. They are characterized by their possessing the aromatic ring in their structure with basic heteroatoms (e.g., imidazole) or the neutral aromatic ring, but containing the basic substituent, polar group, and alkyl chain. Replacing imidazole moiety with the furan ring, weakens the strength of the drug. Introducing the furan cyclic structure with a dimethylaminomethyl substituent as a side chain, yields a highly potent compound named ranitidine. A similar effect in activity was achieved as a result of introducing a plane thiazole ring, which is substituted with the same dimethylaminomethyl group, which contributed to a drug called nizatidine (Figure 5).

In both of these substances, the polar nitro group decreased the lipophilicity of the compounds. Ranitidine is an H_2_ histamine receptor antagonist. It inhibits gastric acid secretion that is stimulated by histamine H_2_, pentagastrin, insulin, caffeine, and food, which explains its use with active peptic ulcers of the stomach and duodenum, and inflammation of the esophagus, which is the consequence of gastroesophageal reflux disease [17,18]. This drug is also used as an agent for preventing the recurrence of duodenal ulcer and stress ulcer formation. By inhibiting the excessive secretion of hydrochloric acid by the parietal cells of the gastric mucosa, nizatidine reduces both the volume of gastric acid secretion and pepsin content thereof. It can also be used in the treatment of dyspepsia [19].

## 6. Anticancer Agents

Advances in the field of oncology have led to the development of many anticancer agents for the treatment of cancer. 

### 6.1. Flutamide and Nilutamide

**Figure 6:**
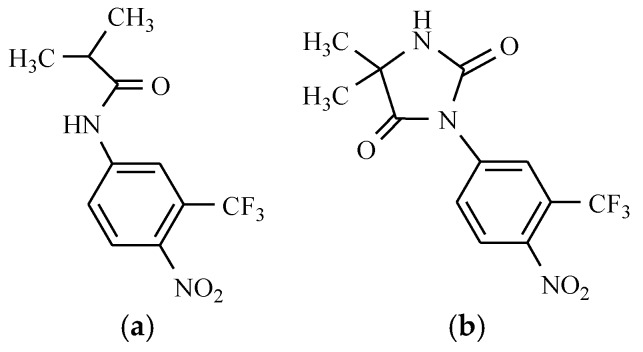
Anticancer drugs: (**a**) Flutamide; and (**b**) Nilutamide.

The antiandrogenic drugs containing the nitro group, flutamide, and nilutamide are widely used in the treatment of carcinoma of the prostate. They are used in combination with agonists of luliberine, resulting in a total androgen blockade [20]. Flutamide and nilutamide contain the nitro group in the benzene ring (Figure 6).

Phenylpropanamide androgen antagonists are among the anticancer active compounds with the nitro substituent. They inhibit transportation to the cell and the binding of dihydrotestosterone in the cell nucleus, which in turn inhibits prostate cell growth and division [13].

Different studies show the principal role of CYP1A2 in the metabolism of flutamide to 2-hydroxyflutamide. The mechanism of flutamide is to block the action of both endogenous and exogenous testosterone, and moreover, it is a potent inhibitor of testosterone-stimulated prostatic DNA synthesis. It is also capable of inhibiting the prostatic nuclear uptake of androgen [21].

The therapeutic effects of nilutamide are overshadowed by the occurrence of adverse reactions, mediated by mechanisms that remain elusive. Studies demonstrate that nilutamide is reduced to its hydroxylamine and amino derivatives and this reduction is oxygen-sensitive [22].

### 6.2. Azathioprine

**Figure 7:**
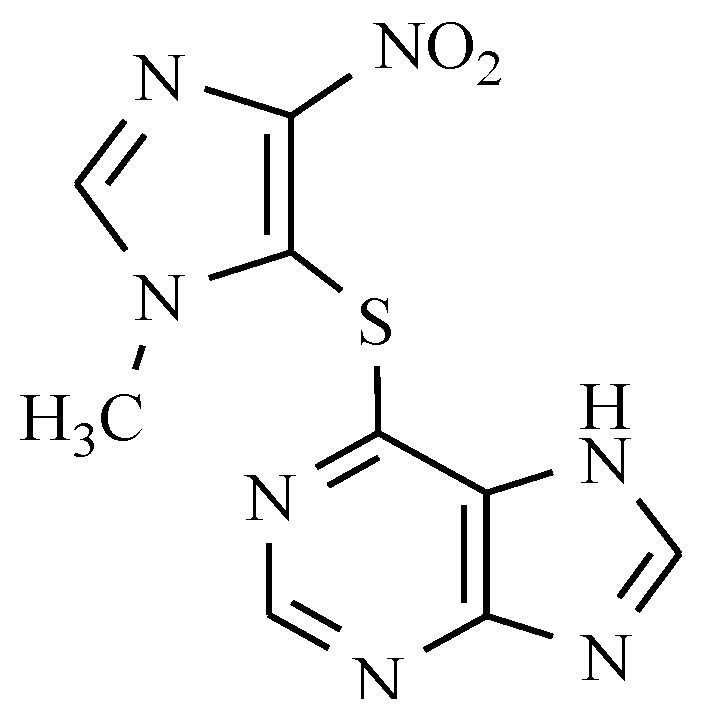
Anti-immunosuppressants: Azathioprine.

Active anti-immune suppressants bearing the nitro group include a pro-drug azathioprine. Azathioprine is a thiopurine that is linked to a second heterocycle (an imidazole derivative) via a thioether (Figure 7).

Azathioprine is converted into 6-mercaptopurine in the body where it blocks purine metabolism and DNA synthesis. The inclusion of thioanalogs of purines in the DNA chain causes DNA helix damage. Azathioprine is used to prevent allograft rejection, severe rheumatoid arthritis, dermatomyositis-muscular, autoimmune chronic active hepatitis, and multiple sclerosis, and also in dermatology [23]. The most recognized uses of azathioprine in dermatology are for immunobullous diseases, photodermatoses, and generalized eczematous disorders [24]. Azathioprine is commonly administered with other drugs, mainly with corticosteroids.

### 6.3. Nitracrine and Rubitecan

**Figure 8:**
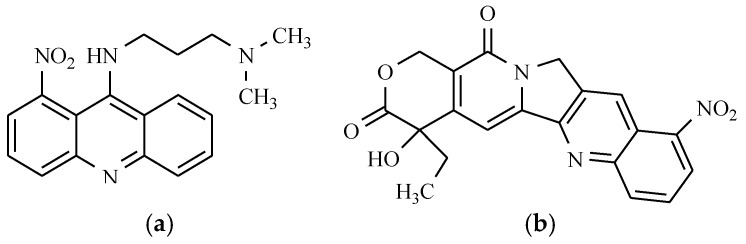
Anticancer drugs: (**a**) Nitracrine; and (**b**) Rubitecan.

Furthermore, nitracrine and rubitecan are anticancer drugs. Nitracrine is an acridine derivative. While rubitecan is a derivative of camptothecin (Figure 8). 

It is approved for the treatment of patients with breast and ovarian cancer because it inhibits the synthesis of RNA. Reducing the nitro group seems to be one of the steps leading to the formation of nitracrine metabolites. It has been found that a lack of the nitro group or its replacement by methyl or halogen at different positions of the acridine ring reduces the activity of the compound obtained. Similarly, the translocation of the nitro group to another position of the benzene ring results in a decrease in activity.

Rubitecan is a derivative of camptothecin, an alkaloid that is extracted from *Camptotheca acuminata* (*Nyssaceae*). Camptothecin has a broad range of anticancer activity, especially against colon cancer and other solid tumors and leukemias. It is not used in therapy due to its high toxicity, which is particularly manifested in hemorrhagic cystitis, gastrointestinal toxicity, and myelosuppression [25]. Rubitecan exists in a 1:1 ratio as 9-nitro-camptothecin and 9-amino-camptothecin. Both of the compounds contain a lactone ring that is required for optimal activity with the carboxylic acid (open ring) forms being significantly less active or inactive. Preclinically, rubitecan has shown activity against a broad spectrum of tumor types in in vitro and in vivo human tumor xenograft models. Unfortunately, the level of activity of an agent in preclinical models has not always translated into similar activity against human tumors in clinical trials. To date, with the exception of pancreatic and possibly ovarian cancer, rubitecan has exhibited disappointing activity against some other solid tumors in relatively small Phase I/II trials; however, it has shown sufficient activity against pancreatic cancer, which is a malignancy [26].

Rubitecan was withdrawn in 2006 because of the existence of serious adverse events in patients that were treated with this substance.

## 7. Antibacterial Drugs

The class of antibacterial nitro drugs includes numerous derivatives of 5-nitrofuran, 2-nitro- and 5-nitroimidazole, 5-nitrothiazole, 5-nitroquinoline, and chloramphenicol.

### 7.1. Derivatives of 5-Nitrofuran

The furan ring system is the basic skeleton of many compounds with biological activities. These moieties are found widely in antibacterial, antiviral, anti-inflammatory, antifungal, anticancer, antihyperglycemic analgesic, anticonvulsant, and other agents. Slight changes in substitution patterns in the furan nucleus cause distinguishable differences in their biological activities. The presence of the nitro group at the C-5 position in the furan ring is essential for antibacterial activity (Table 2 and Table 3). These drugs act as bacteriostatic and antiseptics, and they also show an antifungal and antiprotozoal effect. Nitrofurans act even in small doses, and they do not cause the formation of resistant strains. They have antibacterial potency against pathogenic both Gram-positive and Gram-negative, such as *Escherichia*, *Klebsiella*, *Enterobacter*, *Salmonella*, *Shigella*, and *Vibrio* genus. On the other hand, they are not effective against infections that are caused by *Proteus sp.* and *Pseudomonas aeruginosa*. 

The most widely used drugs from this class are nitrofurantoin, furazidin, nifuroxazide, furazolidone or nifurtoinol, nitrofural, nifurzide, and nifuratel. Nitrofurantoin is a substance that is active against the majority of microorganisms causing urinary tract infections, especially with *E. coli*. Furazidin is a nitrofurantoin analog that has the stronger effect than its parent compound on Gram-positive and Gram-negative bacteria, and also against sulfonamide and some antibiotic-resistant pathogenic strains. Furazidin activity increases in acidic urine. The higher the pH value, the more its effectiveness decreases. Furazidin is used in both acute and chronic urinary tract infections (e.g., suppurative inflammation of renal pelvis, inflammation of prostate and bladder). Moreover, it can be useful in the long-term prevention of infections [27,28,29], and locally for irrigation of wounds, burns and abscesses [2]. Effective therapeutics in bacterial and protozoal diseases of the gastrointestinal tract are nifuroxazide and furazolidone as they are active against *Escherichia, Shigella*, *Salmonella*, and *Klebsiella*. 

These drugs are used in acute and chronic bacterial diarrhoea (also in infants), and during inflammation of the colon and small intestine. Furthermore, nifuroxazide exhibits antiseptic potency. On the other hand, furazolidone is an active agent in trichomonas infection and vaginal thrush [30]. Nihydrazone, nifuroxime, and nifuratel show both antibacterial and antiprotozoal activity. Nifuratel is considered as the alternative to metronidazole, as it has a similar effect on protozoa (*Trichomonas* and *Giardia lamblia*) and *Gardnerella*, with no effect on lactobacilli. Nifuratel is provided with an inhibitory effect on the growth of strains of *Atopobium*, which are strongly associated with bacterial vaginosis, but are resistant to metronidazole [31]. Nifuratel is also effective against *Candida albicans* infections because it damages the structures of enzymes Hwp 1 (Hyphal wall protein 1) and also the cytoplasmic membrane of fungi. By combining methylmercadone with nystatin, a full antibacterial effect is achieved, which determines the broad spectrum of activity, including *Chlamydia*, *Trichomonas vaginalis*, anaerobes, and aerobic Gram-positive and Gram-negative bacteria.

The group of drugs with a 5-nitrofuran moiety also includes nitrovin, which in addition to antimicrobial properties, is also used in veterinary medicine as a growth promoter [32].

Among the derivatives of 5-nitrofuran, other preparations with antibacterial activity should also be emphasised, namely: nifurthiazole, nifurvidine, nifuralide, and nifuratrone, which are used for controlling *Salmonella choleraesuis* in swine, nifurethazone, nifurimide, nifurizone, and nifurmazole. Nifurpipone, nifurtoinol, nifuraldezone, and nifuradene are used in the treatment of urinary tract infections.

#### 5-Nitrofuran Derivatives’ Mechanism of Action

**Scheme 1:**
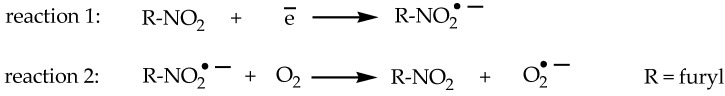
The red-ox reactions of 5-nitrofurans.

The mode of action of 5-nitrofuran analogues is based on red-ox biotransformation. The active moiety is 5-nitro-2-furyl, which can be activated by a biological reduction of the nitro to the hydroxylamino group. These compounds must undergo activation before mediating its cytotoxic effects [33]. These are reactions are catalyzed by nitroreductase (NTR) enzymes. Based on their cofactors, oxygen sensitivities, and product profiles, NTRs can be broadly divided into two groups (reaction 1 and 2, Scheme 1). The ubiquitous oxygen-sensitive type II NTRs are flavin (flavin mononucleotide [FMN] or flavin adenine dinucleotide [FAD]) binding NAD(P)H-dependent enzymes that mediate the 1*e*^−^ reduction of the nitro substrate to form a nitro radical anion (reaction 1, Scheme 1). In an aerobic environment, this radical undergoes futile cycling, resulting in the formation of superoxide anions and the regeneration of the parent nitro compound (reaction 2, Scheme 1). Free radicals can readily react with cellular macromolecules, and they are directly responsible for antibacterial action. As a result, lipids oxidation, cell membrane damages, enzyme inactivation, and, finally fragmentation of the DNA sequence is observed. 

### 7.2. Derivatives of 2-Nitro-, 5-Nitroimidazole and 5-Nitrothiazole

**Figure 9:**
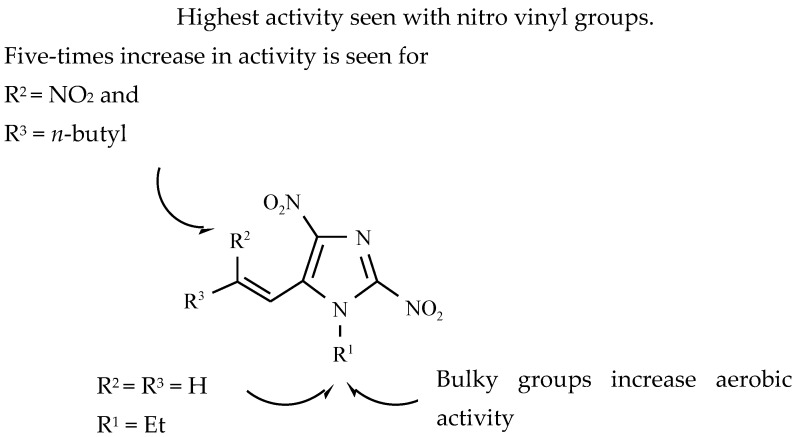
Structure-activity relationships of 2-nitroimidazoles [34].

The discovery of azomycine (2-nitroimidazole) and the confirmation of its biological activity directed against both Gram-positive and Gram-negative and anaerobic microorganisms resulted in increased interest in this group of chemicals. 2-Nitroimidazoles were the first class of nitroimidazoles with reported anti-tubercular activity. A vast array of compounds belonging to this class substituted at 1- and 5-positions was screened against Gram-positive and Gram-negative bacteria, as well as fungi. Taking into account the structure-activity relationship, it should be stated that an increase in lipophilicity at the 5-position of the 2-nitroimidazoles increased the antimicrobial activity of Gram-positive bacteria, including Mycobacterium tuberculosis (Figure 9) [34].

Derivatives of 5-nitroimidazole (azanidazole, ipronidazole, metronidazole, tinidazole, nimorazole, ornidazole, and many others) (Table 4) and 5-nitrothiazole (e.g., niridazole and nitazoxanide) have two-way activity [35]. This strictly concerns anaerobic bacteria and protozoa. In some countries, azanidazole is also approved for the treatment of trichomoniasis. Ipronidazole is mainly used for the veterinary purposes of combating histomoniasis in turkeys and dysentery in pigs. Derivatives of azomycine damage DNA by forming complexes or terminating the thread. Under such anaerobic conditions, the transformation of the reactive metabolites attacking the DNA results in bactericidal activity.

Nitroimidazoles are weakly basic compounds, moderately lipophilic with a low molecular weight, making it easy to penetrate cell membranes and allowing for almost complete absorption into the blood circulation system. In the gastrointestinal tract, they are absorbed quickly, but at different rates. 5-Nitroimidazoles are readily oxidized in the liver (at the C-2 position of the imidazole ring) to hydroxy, acetyl, and carboxylic derivatives, and then they are subjected to conjugation with glucuronic acid and sulphuric acid. Metronidazole shows bactericidal activity against the most important anaerobic microorganisms, from the clinical point of view, including types of *Bacteroides*, *Fusobacterium*, *Megasphaera*, and *Clostridium*, sometimes *Peptococcus*, *Peptostreptococcus* and *Veillonella*, as well as some of the spirochetes. In contrast, the bacteria of the genus *Propionibacterium* and *Actinomyces* are usually resistant to metronidazole. These are used to treat many bacterial infections, especially in gynecology [36], and dentistry, as well as gastric ulcers and duodenal ulcers caused by *Helicobacter pylori*. It was found that metronidazole and tinidazole exhibit a high degree of activity in the treatment of bacterial infections in combination with clarithromycin. Tinidazole is a drug acting against the bacteria of *Gardnerella*, *Propionibacterium*, *Eubacterium*, *Campylobacter*, *Actinomyces*, and *Spirochetes*. Nimorazole, however, is used in acute necrotizing ulcerative stomatitis, and non-specific inflammation of the vagina (Vincent inflammation) [2] and in the control of amebiasis [37]. Ornidazole is applied in the treatment of infections that are caused by susceptible anaerobic bacteria and preventing the disease in the perioperative period. Moreover, it is used in the treatment of rheumatism [38].

### 7.3. Antituberculotic Activity of Nitroimidazole Derivatives

**Figure 10:**
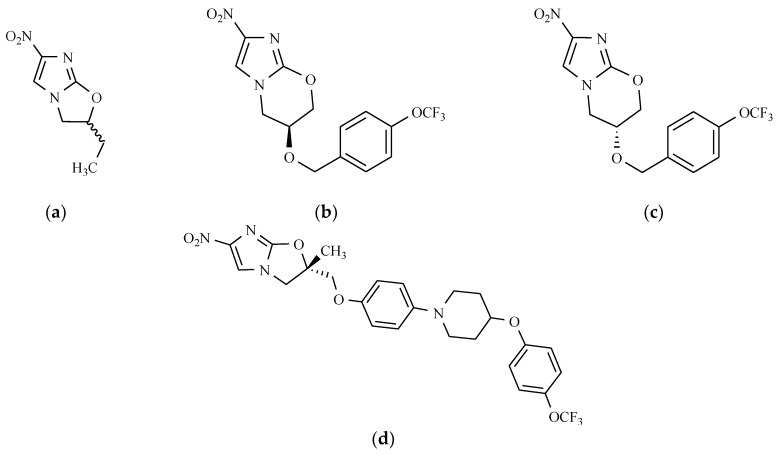
The chemical structure of antitubertulostatic nitroimidazoles: (**a**) CGI-17341; (**b**) (S)-PA-824; (**c**) (R)-PA-824; and (**d**) (S)-OPC-67683.

In 1990, it was discovered that some derivatives of bicyclic nitroazoles, named nitroimidazo[2,1-*b*]dihydrooxazole, might have antituberculotic activity [39]. The leading substance from this series was 2-ethyl-5-nitroimidazo[2,1-*b*]-2,3-dihydrooxazole, designated as CGI-17341 (Figure 10). The results of biological testing showed that the tuberculostatic activity of the compound CGI-17341 was comparable to that of isoniazid (INH) and rifampicin (RIF), which are first-line drugs, and was higher than the activity of antibiotics, such as streptomycin and ciprofloxacin [35]. Furthermore, CGI-17341 showed no cross-resistance with INH and RIF. In a further study, some observations were made regarding the relationship between biological activity and the presence of structural elements in the molecule. It was found that the introduction of a halogen atom in position 2 of the imidazolyl-oxazole system resulted in a 16-fold increase in in vitro activity. The presence of the phenyl ring, as a substituent on the same carbon atom, induced a two-fold increase in tuberculostatic activity, while the long alkyl chain at C-2 decreased potency in vitro. It was also observed that the derivatives with the nitro group in the 5-position of the imidazole ring are two to two thousand times less active (depending on the nature of the substituent at C-2) than isomers of 4-nitroimidazole [40]. The tuberculostatic action mechanisms of compound OPC-67683 (Delamanid) (Figure 10) and isoniazid are very similar and they involve inhibition of mycolic acid synthesis—the main components of the cell wall of *M. tuberculosis*. The difference in behavior between nitroimidazodihydrooxazole and INH is that OPC-67683 is an inhibitor of methoxy- and ketomycolic acids, whereas isoniazid inhibits the formation of all types of these particular fatty acids [41]. OPC-67683 is a prodrug. It is activated by one of the *M. tuberculosis* enzymes, Rv3547, which reduces the nitro group. It is active against strains resistant to rifampicin (RIF), ethambutol (ETH), pyrazinamide (PZA), isoniazid (INH), and streptomycin (SM). OPC-67683 is not mutagenic, and the duration of the therapy can be reduced to two months. It was approved for the European market in 2014 [42]. Only *R* enantiomer is active against mycobacteria. Another extremely promising potential tuberculostatic drug from the group of bicyclic derivatives of nitroimidazoles is a compound signed as PA-824. Interestingly, only *S* enantiomer has tuberculostatic activity. What is more, both chiral forms of PA-824 are active against *Leishmania donovani*, which is the causative agent of visceral leishmaniasis. In leishmania-infected macrophages, (*R*)-PA-824 is even six-fold more active than (*S*)-PA-824 [43]. Chemically, it is a substance with the structure of condensed nitroimidazooxazine (Figure 10) [44].

**Scheme 2:**
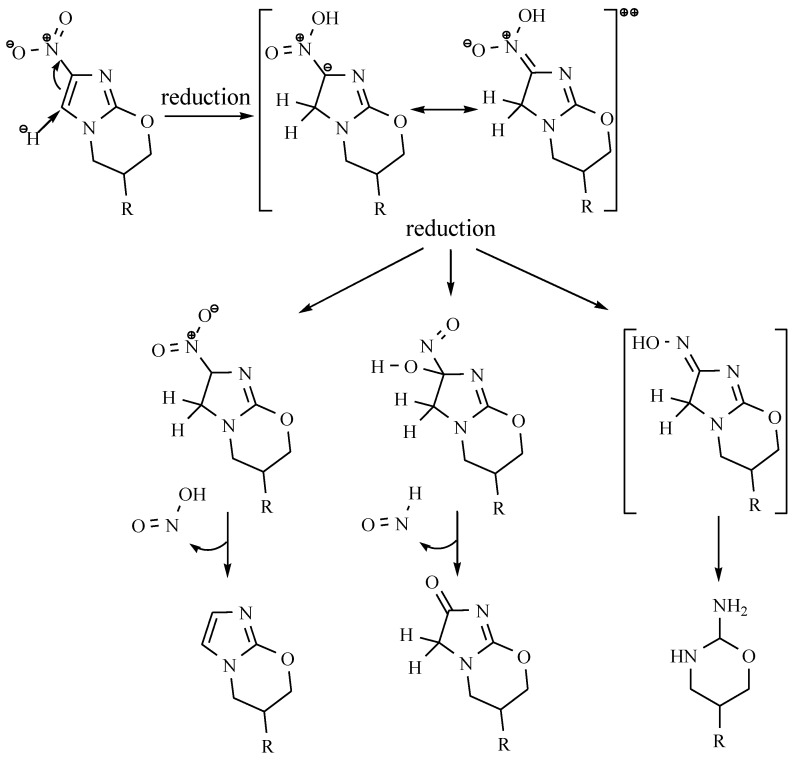
The proposed mechanism for the anticubercular activity of PA-824 [46].

Tests in vitro confirmed its high activity against tuberculosis bacteria, even against strains that are resistant to other drugs. An essential advantage of this compound is also found not to be cross-resistant to other tuberculostatic drugs [45]. Tests in vivo confirm its activity against non-replicated bacteria. The mechanism of action of PA-824 is not fully compprehended, but presumably, it might rely on the creation of radicals that can damage the DNA of *M. tuberculosis*. It has also been noted that, like OPC-67683, PA-824 inhibits the synthesis of mycolic acids and protein biosynthesis [45]. This compound is a prodrug that is activated inside the cell. The mechanism of antitubercular activity is complex and it depends on the enzyme nitroreductase Ddn (Rv3547) [46]. This enzyme produces a biochemical reduction in three directions, which provides three different products. One of the metabolites is des-nitroimidazole. Its formation is closely related to the simultaneous evolution of reactive nitrogen compounds. PA-824 activity in anaerobic conditions is associated with a large number of released nitric oxide (NO) molecules during reduction, which are toxic to bacteria (Scheme 2).

The success of animal studies paved the way for it to be tested in humans. Pharmacokinetic studies of PA-824 in healthy organisms in single as well as multiple-dose studies have shown that the drug is easily absorbed, has good oral bioavailability, and it is safe and well tolerated, with no serious adverse effects [34]. In the coming years, PA-824 could become the primary drug that is used to treat tuberculosis. Currently, it is in late-stage clinical trials. 

Initial structure–activity relationship (SAR) studies have revealed that the replacement of the oxygen atom at the C(9) position of PA-824 with a methylene group results in the loss of antitubercular aerobic and anaerobic activities [47]. However, the 9-position oxygen of the oxazine ring of PA-824 can be replaced by either nitrogen or sulphur, with no significant reduction in MIC value in aerobic conditions, in comparison with the MIC of the parent nitroimidazooxazine [48].

### 7.4. Quinoline Derivatives

**Figure 11:**
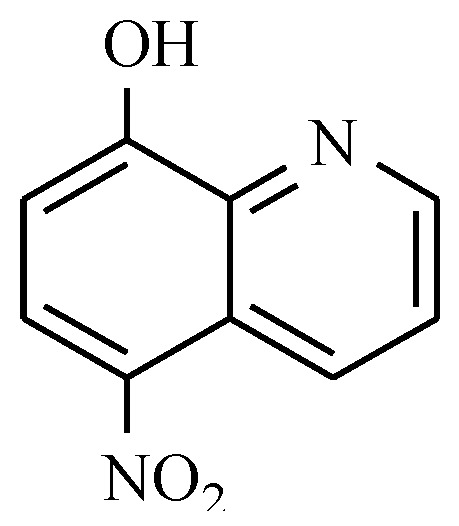
The chemical structure of nitroxoline.

Those drugs containing nitro antibacterial substances also include compounds that are derived from quinoline. Among these drugs with a broad range of antibacterial activity are nitroxoline [49] (Figure 11) and nifuroquine (see: Table 2), which contains the 5-nitrofuran ring. 

They show substantial antibacterial activity towards microorganisms forming a film layer, Gram-negative, Gram-positive bacteria, and additionally against *Candida albicans*. It is used in both acute and chronic urinary tract infections [2]. The established urinary antibiotic nitroxoline has recently enjoyed considerable attention, due to its potent activities in inhibiting angiogenesis, inducing apoptosis and blocking cancer cell invasion. These features make nitroxoline an excellent candidate for anticancer drug repurposing [50]. The addition of a 2-(ethylamino)acetonitrile group to nitroxoline at position 7 significantly improves its pharmacological characteristics and its potential for use as an anti-cancer drug [51].

### 7.5. Chloramphenicol and Its Derivatives

**Figure 12:**
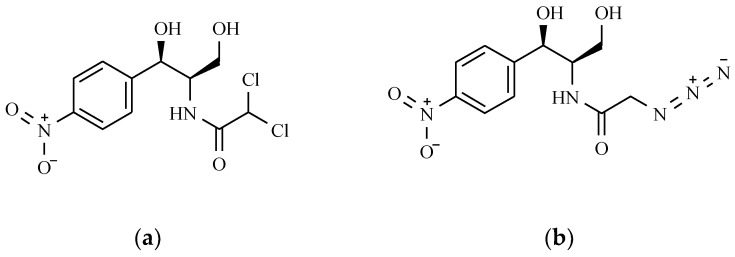
The chemical structure of chloramphenicol (**a**) and its derivative—azidamphenicol (**b**).

The structure of chloramphenicol and its derivative, azidamphenicol (Leukomycin, Posifenicol, Thilocanfol), is presented in Figure 12. 

#### The Mechanism of the Antibacterial Action of Chloramphenicol

**Scheme 3:**
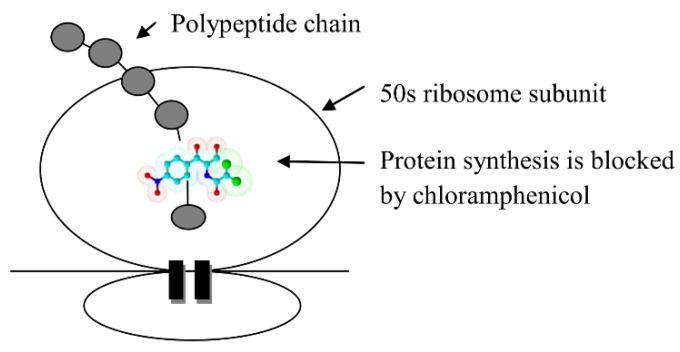
The mechanism of the antibacterial action of chloramphenicol.

It was originally isolated from the bacterium *Streptomyces venezuelae*, by David Gottlieb. It appeared in clinical practice in 1949, under the trade name Chloromycetin. Azidamfenicol has a similar profile to chloramphenicol. Both drugs have been useful in treating ocular infections that were caused by some bacteria, including *Staphylococcus aureus, Streptococcus pneumoniae*, and *Escherichia coli*. They are not effective against *Pseudomonas aeruginosa*. Currently, these drugs are obtained only synthetically [52,53]. The mechanism of the antibacterial action of chloramphenicol is based on blocking protein synthesis in ribosome (Scheme 3). 

Chloramphenicol has a bacteriostatic effect against *Staphylococcus* and Gram-negative bacteria of the family *Enterobacteriaceae*, and bactericidal against *Haemophilus influenzae*, *Streptococcus pneumoniae*, and *Neisseria meningitidis*. Because of the risk of severe side effects, this drug is used in exceptional disease states, as an antibiotic alternative for the treatment of life-threatening infections with *Pseudomonas* and *Haemophilus influenzae* (meningitis), brain abscesses, anaerobic infections (destructive lung infections, brain abscess, pelvic abscess), typhoid fever, brucellosis, rickettsiosis, and tularemia.

### 7.6. Others Antibacterial Nitro Drugs

**Figure 13:**
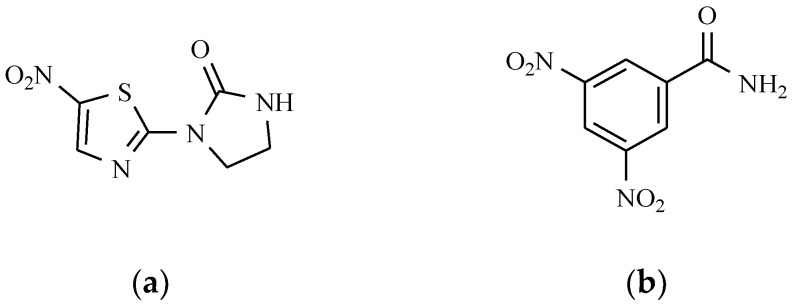
The chemical structures of 5-nitrothiazole: (**a**) Niridazole, and (**b**) Nitromide.

Among preparations containing a derivative of 5-nitrothiazole as the active ingredient, antibacterial and particularly antiprotozoal properties are exhibited by niridazole, which is an active chemotherapeutic agent in the treatment of schistosomiasis (Figure 11). The group of antibacterial drugs also includes an amide of benzoic acid with two nitro groups, e.g., nitromide (Figure 13). This drug has only found application in veterinary therapy as coccidiostatic [3].

## 8. Anthelmintics

**Figure 14:**
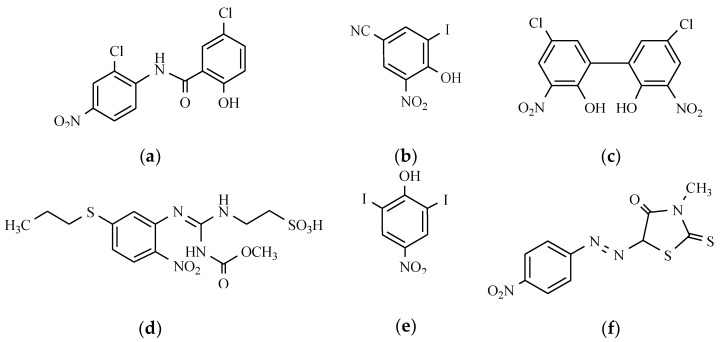
The chemical structure of anthelmintics: (**a**) Niclosamide; (**b**) Nitroxynil; (**c**) Niclofolan; (**d**) Netobimin; (**e**) Disophenol; and (**f**) Nitrodan.

The parasites that cause infections of the gastrointestinal tract in our latitude come from the two families: roundworms (*Nemathelminthes*) and tapeworms (*Plathelminthes*), which include tapeworms (*Cestoda*) and flukes (*Trematoda*) [30]. Drugs used for the control of parasitic diseases belong to different chemical groups, and their mechanism of action also varies. The drug of choice for the treatment of taeniasis, and which contains the nitro group in their structure, is niclosamide (Figure 14). It is used in the taeniasis of the gastrointestinal system that is caused by *Taenia solium*, *Taenia saginata*, *Diphyllobothrium latum*, *Hymenolepis nana*, *Dipylidum caninum*, and *Fasciolopsis buski* [54]. Sometimes niclosamide is used for the treatment of the mollusc *Bulinus* in water reservoirs in the prevention of schistosomiasis endemics [1]. The beneficial effects of the therapy with niclosamide caused the use of other pharmacological agents, such as nitroxynil, which has a strong effect, especially in the mature form of *Fasciola hepatica* and niclofolan (Figure 14). Drugs containing the nitrobenzene ring and exhibiting activity against flatworms are mainly used as veterinary drugs, include netobimin, which belongs to probenzimidazole, nitrodan, and disophenol (Figure 14). Disophenol, for instance, is a nitrophenolic antiparasitic compound that is very useful in controlling several helminth-induced infections in dogs, cats, birds, sheep, and bovines, among other animals. Probenzimidazole is converted in the digestive tract of the host into an active compound belonging to benzimidazole. Netobimin is converted into albendazole.

The group of aromatic nitroisothiocyanates includes nitroscanate and a compound with the INN name Amoscanate, which were used in combating flukes and duodenum hookworms. Unfortunately, widespread use of Amoscanate in veterinary medicine is limited because of its high hepatotoxicity. 

**Figure 15:**
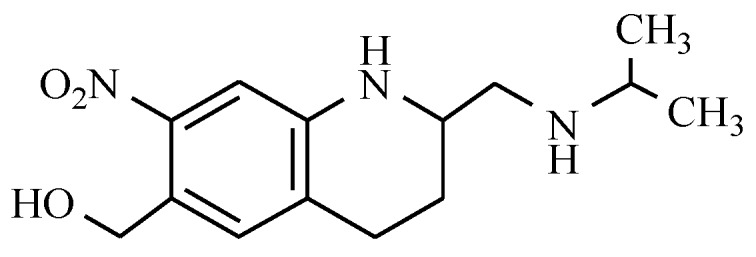
The chemical structure of oxamniquine.

Oxamniquine is a drug that is used to treat infections of fluke causing a disease called schistosomiasis. (Figure 15).

This compound is useful in the treatment of acute and chronic schistosomiasis, which are induced in particular by various forms of *Schistosoma mansoni* and *Schistosoma intercalatum*. Perhaps one of the mechanisms of their action is to cause worms to shift from the mesenteric veins to the liver where the male worms are retained and are subsequently destracted. Females remaining in the mesenteric veins are unable to release eggs [2]. Because of the broad activity against schistosomiasis, niridazole (see Figure 13) is also used, which is characterized by the antibacterial and antiprotozoal potencies, as discussed previously [55]. In contrast to oxamniquine, niridazole has a pharmacological effect on *Schistosoma haematobium* flukes, and also a lesser effect on *Schistosoma mansoni* and *Schistosoma japonicum*. This drug is also effective against *Dracunculus medinensis* nematodes, and in the treatment of amebiasis invasion in cases of resistance developed to other treatment, or the inability to use it. It also shows beneficial effects in the treatment of *Onchocerciasis*, cutaneous leishmaniasis, and infections that are caused by fleas *Tunga penetraus* [2].

**Figure 16:**
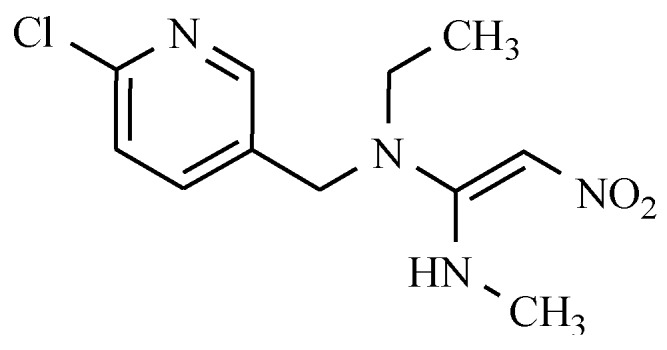
The chemical structure of nitenpyram.

Veterinary medicine uses a halogenated derivative pyridine, called nitenpyram (Figure 16), which is intended for controlling external parasites of dogs and cats, such as fleas. Nitenpyram is neonicotinoid (new nicotine-like insecticides) that binds particularly well in the central nervous system of insects, causing their rapid death.

### The Mechanisms of Action of Anthelmintic Drugs

**Scheme 4:**
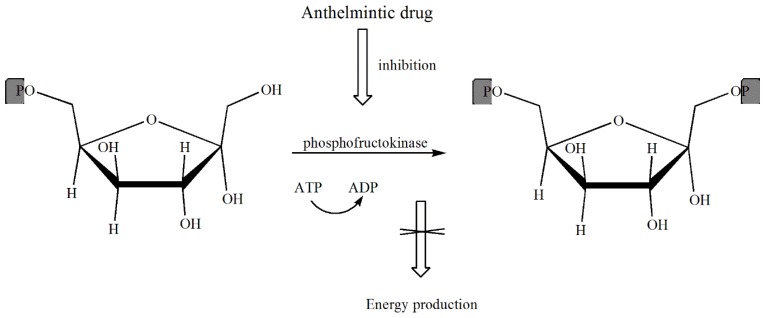
The mechanism of action of anthelmintic drugs.

One of the mechanisms for the action of anthelmintic drugs is the inhibition of energy processes in the parasite’s body. Drugs acting as anthelmintics interfere with the metabolism of carbohydrates, causing the incomplete oxidation of substrates in worms. The primary source of energy production in worms is the process of glycolysis, catalysed by phosphofructokinase. This enzyme facilitates phosphorylation of fructose 6-phosphate to fructose-1,6-diphosphate. The sensitivity of the fructokinase, e.g., in Schistosoma to inhibitors is much greater than the corresponding enzyme in mammals. Blocking the fructokinase that is involved in glycolysis causes an accumulation of the substrate, which is fructose-6-P (Scheme 4).

## 9. Antiprotozoal Drugs

Pathogenic protozoa belong to many groups of unicellular organisms, among which the following stand out: dinoflagellates, rugrats, and trypanosomes. In many parts of the world, protozoal infections are the most common causes of diseases. In Poland, however, the most frequently occurring ones are trichomoniasis that are caused by *Trichomonas vaginitis* (*Trichomonas vaginalis*), giardiasis caused by *Lamblia intestinalis* and toxoplasmosis caused by protozoa *Toxoplasma gondii*. A rarer case is amebiasis (amoebic dysentery), which is caused by the amoeba *Entamoeba histolytica* occurring in the gastrointestinal tract [30].

### 9.1. Nitroimidazole Derivatives

**Figure 17:**
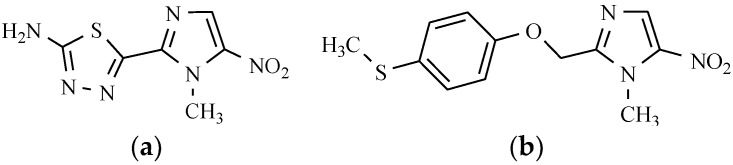
5-Nitroimidazoles as antiprotozoal drugs: (**a**) Megazol; and (**b**) Fexinidazole.

Megazol, which is a 5-nitroimidazole derivative containing the thiadiazole ring (Figure 17), is hugely effective against *Trypanosoma brucei* and *Trypanosoma cruzi*. Another 5-nitroimidazole derivative is fexinidazole (Figure 17), demonstrating potent in vitro and in vivo activity against trichomonas, *Entamoeba histolytica*, *Trypanosoma cruzi*, and *Trypanosoma brucei* [56].

The biologically relevant active metabolites in vivo are the sulfoxide and sulfone of the compound that is mentioned above. In another study, fexinidazole was established as a promising candidate for the acute and chronic stages of the African human trypanosomiasis. In 2009, this substance became the first new trypanosomatid illness clinical candidate for three decades. Currently, it is undergoing Phase III clinical trials [57].

Now, the drugs of choice for the treatment of infections that are caused by *Trichomonas vaginalis* are analogues of 5-nitroimidazole. The most essential connections from this group include metronidazole, ornidazole, tinidazole, nimorazole and azanidazole (see: Table 3). These drugs act against *Entamoeba histolytica*, *Lamblia intestinalis* and the majority of absolute anaerobic bacteria, as mentioned before. Nitroimidazoles easily penetrate the single-celled organisms of protozoans. The action of these drugs is associated with a reduction in the nitro group and the formation of cytotoxic agents for protozoa. The products of this reduction are formed within cells involving ferredoxin, which is the electron transport protein and only occurring in organisms with anaerobic metabolism or those deficient in oxygen. The source of electrons that are needed for the reduction may also be endogenous substances, such as reduced nicotinamide adenine dinucleotide phosphate (NADPH). The reduced form of the drug acts on microbial DNA by breaking the chain and then causes a protozoan cell damage. 

Metronidazole and other derivatives of the 5-nitroimidazole administered orally are readily absorbed from the gastrointestinal tract. Moreover, they can be used vaginally and percutane ointment, thus also reaching high levels in the blood. In addition to treating infections that are caused by these protozoa, metronidazole is also used in the control *Giardia lamblia*, *Gardnerella vaginalis*, *Blastocystis hominis* [2]. 5-Nitroimidazole derivatives include secnidazole (see: Table 3), which has potent activity as a protozoonicidal. It is used in intestinal and hepatic amebiasis and trichomoniasis. It was also found that a combination of the nitroimidazole derivatives, i.e., metronidazole, tinidazole, or secnidazole with amphotericin B has high activity against *Candida albicans*. 

Derivatives of the 5-nitroimidazole are widely used in veterinary medicine because they are useful agents for the treatment of protozoa invasion and infections that are caused by anaerobic bacteria. However, due to their genotoxic and carcinogenic potential, their use in food-producing animals or products that are intended for human consumption is prohibited in many countries. Nitroimidazoles, which in the past were registered in the European Union (EU) as veterinary drugs or food additives include metronidazole, dimetridazole, ronidazole, and ipronidazole. They were used because of their effectiveness in treating histomoniasis found in the poultry, which is caused by the flagellate *Histomonas meleagridis* [58].

### 9.2. 5-Nitrofuran Derivatives

**Figure 18:**
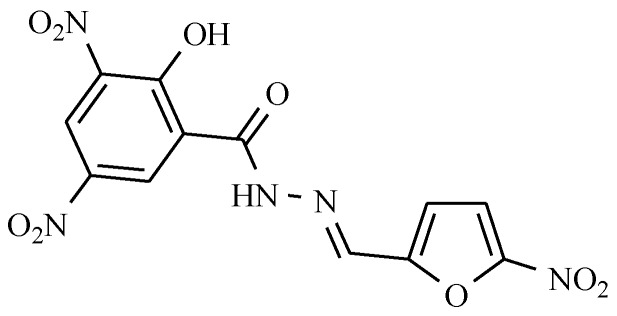
Nifursol as anti-trypanosomiasis drug.

This group includes nifurtimox (see: Table 2), which is used in the treatment of American trypanosomiasis, a disease occurring mostly in the rural areas of the South America carried by bugs [30,59] and nifursol, which acts against histomoniasis (Figure 18). 

Nifurtimox was used in the treatment of acute Chagas disease when it was identified by Bayer in in vitro screens against *Trypanosoma cruzi*. For many years, nifurtimox was considered to be the front-line therapy for this indication. Nifurtimox is also effective against *Trypanosoma brucei gambiense* infection. 

### 9.3. 2-Nitroimidazole Derivatives

**Figure 19:**
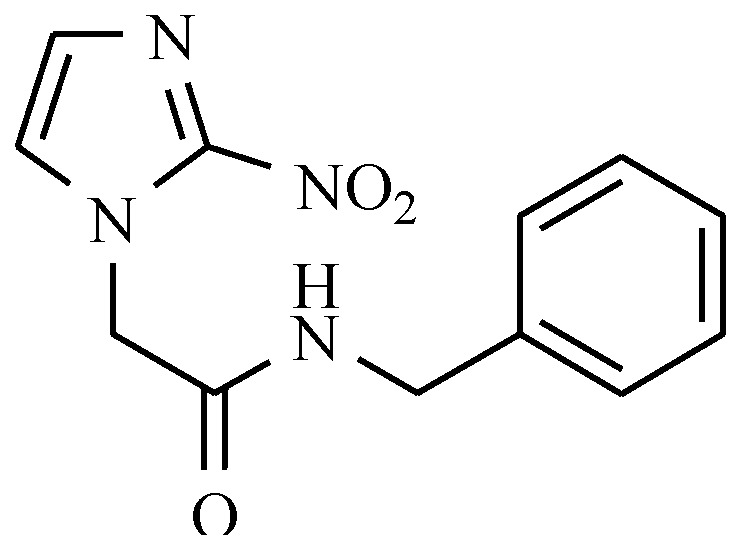
Benznidazole.

The current drug of choice for acute-stage Chagas disease is benznidazole, which is a derivative of 2-nitroimidazole (Figure 19). Benznidazole has been used in the treatment of Chagas disease for about 40 years. Unfortunately, a range of serious side effects is associated with its use, including dermatological reactions, agranulocytosis, and polyneuropathy [56].

### 9.4. 5-Nitrothiazole Derivatives

Aminitrozole, nithiazide, nitazoxanide, tenonitrozole, and tizoxanide are drugs from the 5-nitrothiazole group, which act as antifungals and antiprotozoals, especially in the treatment of trichomoniasis (Table 5). 

Formulations containing nitazoxanide are indicated in the treatment of diarrhea that is caused by *Cryptosporidium parvum* or *Giardia lamblia*. Nitazoxanide is an anti-infective prodrug, which very quickly converts to an active metabolite tizoxanide. The parent nitazoxanide is not detected in plasma. Tizoxanide is active against anaerobic bacteria, protozoan parasites, and viruses [60].

### 9.5. Others Antiprotozoal Drugs 

**Figure 20:**
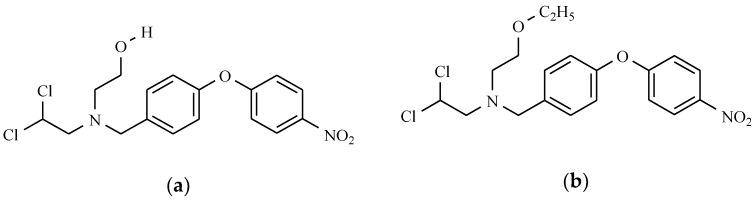
Nitrophenyl derivatives as antiprotozoal drugs: (**a**) Clefamide; and (**b**) Etofamide.

Etofamide and clefamide are other antiprotozoal drugs that are effective in combating intestinal amebiasis. These are compounds that are derived from dichloroacetamide containing a nitrophenoxyphenyl substituent as a side moiety (Figure 20).

## 10. Radiosensitizers

Knowledge of oxygen’s effect led to the development of compounds that mimic its radiosensitizing property. The radiosensitizing capabilities of the hypoxic cell sensitizers have been found to connect with electron affinity [61]. 2-Nitroimidazoles play a great role as bioreductive markers for tumour hypoxia and as radiosensitizers [35]. It is known that 2-nitroimidazoles (e.g., misonidazole, etanidazole) are more active than 5-nitroimidazoles (e.g., metronidazole, nimorazole) as hypoxic cell radiosensitizers. The differences in their activity in vitro correlate most closely with alterations in redox properties, especially in their electron-affinity [62].

The mechanism of action of this class of sensitizers is based on the “oxygen fixation” hypothesis [63]. They fix radiation damage by preventing the chemical restitutions of free radicals. Misonidazole has been observed to deplete -SH groups in cells, and to inhibit both glycolysis and the repair of potentially lethal radiation-induced cellular damage [64]. Clinical trials with misonidazole have shown undesirable effects, such as peripheral neuropathy, convulsions, and encephalopathy, and therefore its use is severely limited [65].

Etanidazole has been analyzed with perspective results in early phase II and III clinical trials. It appears to be less toxic to CNS tissue than misonidazole and crosses the blood-brain barrier in limited quantities. A phase III study of this agent showed increased survival in the two-year local control in N0 and N1 disease with 55% in the etanidazole arm and 37% in the radiation-alone arm [66].

Nimorazole is a member of the same structural class as metronidazole. However, it is less toxic, thus allowing for higher doses to be administered. A phase III study of nimorazole versus a placebo in subjects with squamous cell carcinoma of the supraglottic larynx and pharynx demonstrated a statistically significant difference regarding improvements in loco-regional control at five-year post-treatment [67]. In phase II, a study of nimorazole was conducted in patients with stage 3 or 4 squamous cell carcinoma of the head and neck who received continuous hyperfractionated accelerated radiation therapy (CHART). It was found that local control rates were higher than in other studies using CHART, suggesting the positive effect of nimorazole.

## 11. Drugs with Others Effects

**Figure 21:**
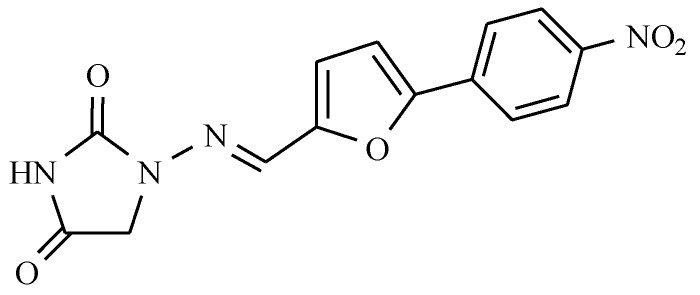
Dantrolene.

Medications that do not belong to any of the above-described families and contain the nitro group are also present on the pharmaceuticals market. One such medicine is dantrolene, whose structure includes the hydantoin system, furan, and p-nitrophenol rings (Figure 21). 

**Scheme 5:**
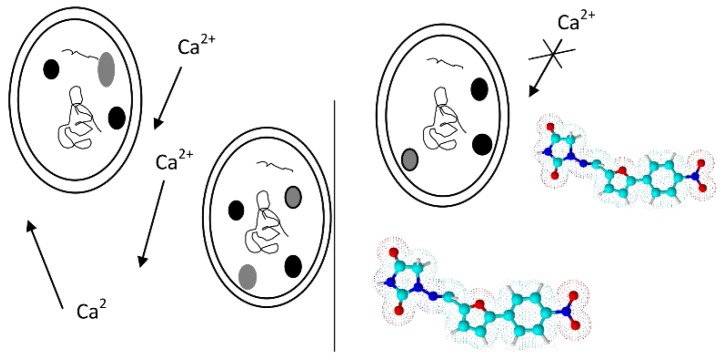
The mechanism of action of dantrolene.

This is a drug acting as a striated muscle relaxant and that distorts the contraction of the muscle cell by inhibiting the intracellular movements of calcium ions [68] (Scheme 5). Dantrolene is used to treat post-stroke spasticity, resulting in the brain, spinal cord injury, cerebral palsy, multiple sclerosis running with paresis, and anesthesia for the prevention and treatment of malignant hyperthermia [69,70].

**Figure 22:**
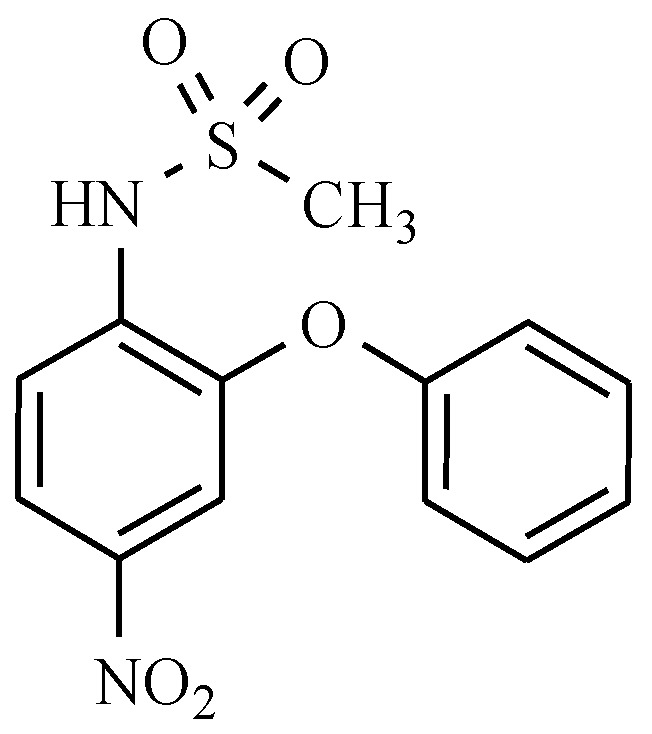
Nimesulide.

Next, nimesulide is a non-steroidal anti-inflammatory drug, analgesic, and antipyretic, belonging to the sulfonanilide group containing a diphenyl ether skeleton substituted with methanesulphonamide and nitro groups (Figure 22).

**Scheme 6:**
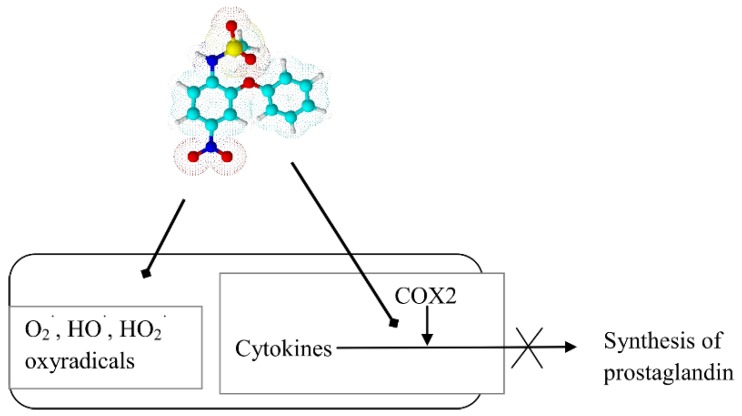
Sites of action of nimesulide in cellular inflammation.

It inhibits the formation of free radical peroxide and it also blocks the activity of specific cyclooxygenase, thereby reducing prostaglandin synthesis (Scheme 6). The indication for nimesulide use is pain that is associated with bone diseases and joint pain, neuralgic, postoperative pain, and trauma [71,72]. Unfortunately, nimesulide had to be withdrawn in many countries due to the serious risk of fatal hepatic disorders. 

**Figure 23:**
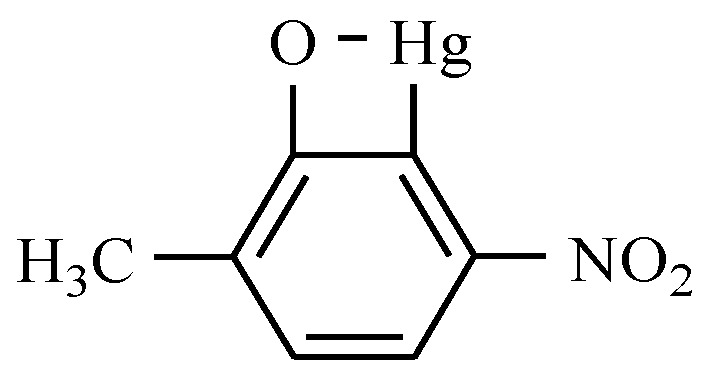
Nitromersol.

Nitromersol is a bicyclic compound that is used as an antiseptic and disinfectant for surgical and dental use (Figure 23). However, the presence of mercury in its structure has led to its limited use.

**Figure 24:**
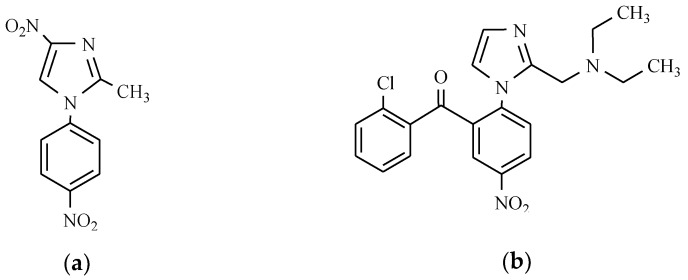
The 4-nitroimidazole derivatives as nootropic: (**a**) Nitrefazole; and (**b**) Nizofenone.

Moreover, two more compounds with the nitro group also exhibit pharmacological activity. For the treatment of alcoholism, nitrefazole, which is a nitrophenyl derivative of 4-nitroimidazole, is used, while nizofenone, which has nootropic activity, is a derivative of nitrobenzophenone comprising a substituted imidazole ring (Figure 24).

**Figure 25:**
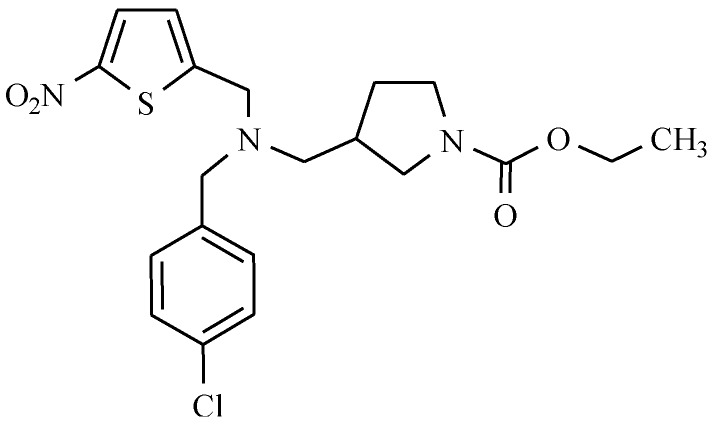
The structure of SR9009.

Among the group of the nuclear receptors family (REV-ERB) activity modulators, a nitro compound called SR9009 can be found (Figure 25). 

It alters the expression of genes that are involved in lipid and glucose metabolism, and therefore, it plays an essential role in maintaining the energy homeostasis [73,74]. Additionally, this substance increases basal oxygen consumption, decreases lipogenesis, cholesterol and bile acid synthesis in the liver, increases mitochondrial content, glucose and fatty acid oxidation in the skeletal muscle, and decreases lipid storage in the white adipose tissue. These observations make SR9009 a promising drug for the treatment of several metabolic disorders [73,74].

## 12. Drugs of the Future

**Scheme 7:**
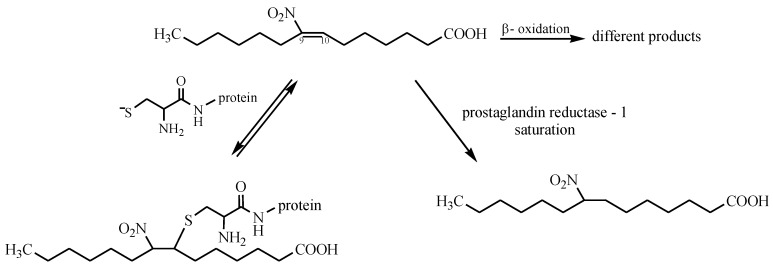
Mechanism of action of nitro-fatty acids.

Among exciting nitro compounds, a group of nitro-fatty acids (NO_2_-FAs) can be found. They are formed in human plasma, cell membranes, and tissue by redox reactions of unsaturated fatty acids with secondary products of NO oxidation, e.g., NO_2_, NO_2_^−^, NO_3_^−^ [75]. This process occurs under physiological conditions—during digestion, where nitration is favored by the low pH of gastric juice in the stomach, and metabolism catalyzed by peroxidases, globins, and nitric oxide autooxidation [76]. However, the correct mechanism in vivo remains unknown [77]. Nitro-fatty acids are transported back into blood circulation in the form of conjugates that are synthesized by covalent adduction with glutathione (GSH) (Michael reaction) [78]. These reactions between proteins and NO_2_-FAs are significant in the context of cell signaling, as this reaction is reversible. Protein adduction by NO_2_-FAs is detected clinically, thus representing a metabolic and redox-sensitive mechanism for regulating protein distribution and function [79]. Other mechanisms are associated with hepatic β-oxidation and double-bond saturation (Scheme 7) [80].

Furthermore, nitro-fatty acids can release nitric oxide [81]. They can be used as the source of NO in the organism. These compounds have full potential in medicine. They exert a long-term cardioprotective effect in experimental models of metabolic and cardiovascular diseases. They reduce lipid accumulation and promote plaque stability in atherosclerosis. As is shown in the literature [82], acute administration of NO_2_-FAs is effective to minimize vascular inflammation in vivo. They act as disruptors of the TLR4 signaling complex in lipid rafts, leading to the resolution of pro-inflammatory activation. Moreover, nitro-fatty acids reduce blood pressure in an angiotensin II infusion model of hypertension. They interfere with angiotensin II signaling, thus causing limited calcium mobilization in vascular smooth muscle cells. This is the main cause of blood pressure reduction [83]. Considerable therapeutic potential is observed for the use of NO_2_-FAs in renal inflammation and kidney diseases. Preliminary studies revealed that treatment with nitro-fatty acids significantly reduces creatinine levels, urinary lipid peroxidation products, and renal inflammation [84]. Nowadays, the active NO_2_-FAs compound, named CXA-10 (10-nitro-oleic acid), undergo phase II clinical trials [84].

## 13. Conclusions

A great deal of research and medical use has revealed the huge potential of nitro compounds to treat different diseases and has eroded the long-held prejudice against them. In many cases, these drugs are essential in combating some types of ailments. There is still a need to search for novel, safe, and effective connections with potential medicinal properties resulting from the presence of the molecule with the nitro group in their structure. This task can be performed by the synthesis of new hybrid combinations of drugs containing the nitro group with fragments of macromolecular structures. During the drug design process, it should be remembered that the inclusion of the nitro group in a molecule alters the physicochemical and electronic properties, and is connected with increased mutagenicity and carcinogenicity [85]. Such hybrids appear in the initial phase of the trial. Treatment should take into account the risk that is associated with toxicity, acquired drug resistance, or cost of production. Currently, it seems that the most promising nitro-compounds with biological activity are nitro-fatty acids. Identification of their metabolism can lead to the discovery of new nitrated unsaturated acids, which are as yet unknown. Work on nitro-fatty acids significantly expanded the knowledge of biological redox processes, which can prove fruitful in the future. 

## Figures and Tables

**Table 1:** Nifedipine derivatives.

General Structure	Derivative	Nitro Group Position	R^1^	R^2^	R^3^
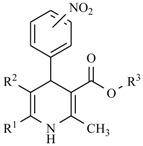	Aranidipine	*ortho*	-CH_3_	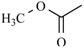	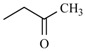
	Barnidipine	*meta*	-CH_3_	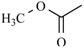	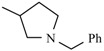
	Benidipine	*meta*	-CH_3_	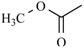	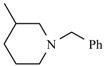
	Cylnidipine	*meta*	-CH_3_	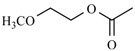	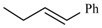
	Efonidipine	*meta*	-CH_3_	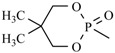	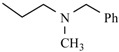
	Nifedipine	*ortho*	-CH_3_	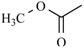	-CH_3_
	Nicardipine	*meta*	-CH_3_	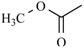	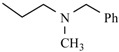
	Nilvadipine	*meta*	-CN	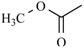	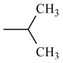
	Nisoldipine	*ortho*	-CH_3_	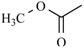	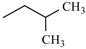
	Nitrendipine	*meta*	-CH_3_	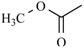	-CH_2_CH_3_
	Pranidipine	*meta*	-CH_3_	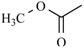	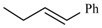
	Nimodipine	*meta*	-CH_3_	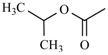	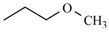
	Manidipine	*ortho*	-CH_3_	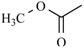	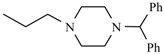
	Falnidipine	*ortho*	-CH_3_	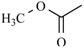	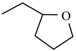

**Table 2:** 5-Nitrofuran analogues with antibacterial activity.

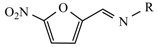		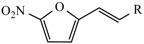	
Compound	R	Compound	R
Nitrofurantoin	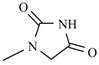	Furazidine	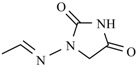
Furazolidone	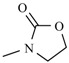	Nifurzide	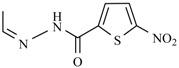
Nifuroxime	-OH	Nitrovin	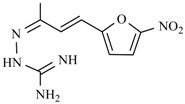
Nifurtoinol	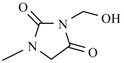	Nifurmazole	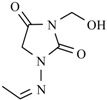
Nifuratel	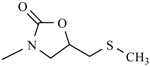	Nifurizone	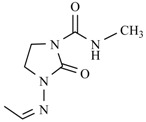
Nifuradene	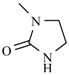	Nifurvidine	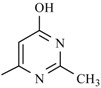
Nifurimide	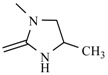	Nifuralide	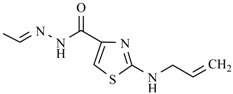
Nifurtimox	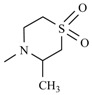	Nifurpirinol	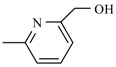
Furaltadone	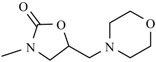	Nifurprazine	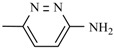
Nifurfoline	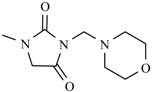		

**Table 3:** 5-Nitrofuran analogues with antibacterial activity.

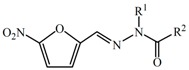			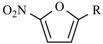	
Compound	R^1^	R^2^	Compound	R
Nifuroxazide	-H	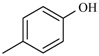	Nifurthiazole	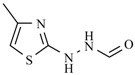
Nifurpipone	-H	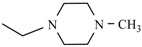	Nifuratrone	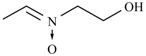
Nifuraldezone	-H	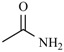	Furazolium	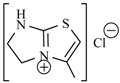
Nifurethazone	-(CH_2_)_2_N(CH_3_)_2_	-NH_2_	Nifuroquine	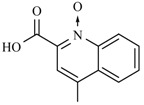
Nitrofural	-H	-NH_2_		
Nidroxyzone	-(CH_2_)_2_OH	-NH_2_		
Nihydrazone	-H	-CH_3_		

**Table 4:** Antibacterial 5-nitroimidazole derivatives.

General Structure	Derivative	R^1^	R^2^
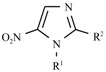	Metronidazole	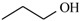	-CH_3_
	Nimorazole	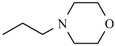	-H
	Ornidazole	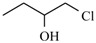	-CH_3_
	Secnidazole	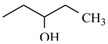	-CH_3_
	Tinidazole	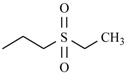	-CH_3_
	Ipronidazole	-CH_3_	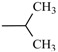
	Azanidazole	-CH_3_	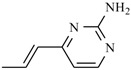
	Megazole	-CH_3_	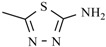
	Propenidazole	-CH_3_	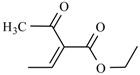

**Table 5:** Antiprotozoal 5-nitrothiazole derivatives.

Main Structure Caption	Derivative	R
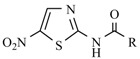	Aminitrazole	-CH_3_
	Nithiazide	-NH-CH_2_-CH_3_
	Tenonitrozole	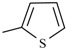
	Tizoxanide	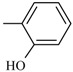
	Nitazoxanide